# Anti-Lymphoma Efficacy Comparison of Anti-CD20 Monoclonal Antibody-Targeted and Non-Targeted Star-Shaped Polymer-Prodrug Conjugates

**DOI:** 10.3390/molecules201119664

**Published:** 2015-11-04

**Authors:** Ondřej Lidický, Olga Janoušková, Jiří Strohalm, Mahmudul Alam, Pavel Klener, Tomáš Etrych

**Affiliations:** 1 Institute of Macromolecular Chemistry, Academy of Sciences of the Czech Republic, Heyrovský Sq. 2, 162 06 Prague 6, Czech Republic; lidicky@imc.cas.cz (O.L.); janouskova@imc.cas.cz (O.J.); strohalm@imc.cas.cz (J.S.); 2 Institute of Pathological Physiology, First Faculty of Medicine, Charles University in Prague, U Nemocnice 5, 128 53 Prague 2, Czech Republic; mahmuddso2004@gmail.com (M.A.); pavel.klener@gmail.com (P.K.); 3 Department of Hematology, Charles University General Hospital in Prague, U Nemocnice 2, 128 08 Prague 2, Czech Republic

**Keywords:** HPMA copolymers, drug delivery systems, doxorubicin, monoclonal antibody, drug targeting

## Abstract

Here we describe the synthesis and biological properties of two types of star-shaped polymer-doxorubicin conjugates: non-targeted conjugate prepared as long-circulating high-molecular-weight (HMW) polymer prodrugs with a dendrimer core and a targeted conjugate with the anti-CD20 monoclonal antibody (mAb) rituximab (RTX). The copolymers were linked to the dendrimer core or to the reduced mAb via one-point attachment forming a star-shaped structure with a central antibody or dendrimer surrounded by hydrophilic polymer chains. The anticancer drug doxorubicin (DOX) was attached to the *N*-(2-hydroxypropyl)methacrylamide (HPMA)-based copolymer chain in star polymer systems via a pH-labile hydrazone linkage. Such polymer-DOX conjugates were fairly stable in aqueous solutions at pH 7.4, and the drug was readily released in mildly acidic environments at pH 5–5.5 by hydrolysis of the hydrazone bonds. The cytotoxicity of the polymer conjugates was tested on several CD20-positive or negative human cell lines. Similar levels of *in vitro* cytotoxicity were observed for all tested polymer conjugates regardless of type or structure. *In vivo* experiments using primary cell-based murine xenograft models of human diffuse large B-cell lymphoma confirmed the superior anti-lymphoma efficacy of the polymer-bound DOX conjugate when compared with the original drug. Targeting with RTX did not further enhance the anti-lymphoma efficacy relative to the non-targeted star polymer conjugate. Two mechanisms could play roles in these findings: changes in the binding ability to the CD-20 receptor and a significant loss of the immunological properties of RTX in the polymer conjugates.

## 1. Introduction

A variety of drug delivery systems (DDS) suitable for improving the biodistribution and biological outcomes of drugs have been developed in the past few decades [1,2]. Among them, conjugates based on *N*-(2-hydroxypropyl)methacrylamide (HPMA) copolymers have shown particular promise as carriers [3]. They improve drug solubility, bioavailability, stability, prolong the circulation time and reduce drug toxicity. Most HPMA-based conjugates have been developed as tumour tissue or tumour cell-specific prodrugs. Tumour-specific drug release is achieved by degradation of specific, enzymatically-cleavable oligopeptide linkers (e.g., the GFLG sequence) via lysosomal enzymes [4,5] or by pH-controlled chemical hydrolysis of linking bonds (e.g., hydrazone bonds, *cis*-aconityl spacers) [6,7]. Moreover, the molecular weight of a polymer conjugate influences the targeting strategy and polymer elimination from the body.

High-molecular-weight (HMW) HPMA-based polymer carriers can be used for the passive accumulation of drugs due to the enhanced permeability and retention (EPR) effect [8,9]. This phenomenon has been demonstrated for HPMA copolymers (e.g., grafted, multi-block or star-shaped polymers) in mice for sarcomas [10], lymphomas [11,12] and other tumours [13].

To effectively target the polymer prodrug, specific moieties with high affinities for the target cell are attached to the conjugate. Since the first polymer DDS, using galactosamine to target liver cells, was published [14], many other tissue or cell-specific ligands have been used as effective targeting moieties, including carbohydrates, lectins, antibodies, antibody fragments and peptides [4].

Highly specific prodrug targeting can be obtained by conjugating monoclonal antibodies (mAbs) with polymer prodrugs. Various targeted mAb-polymer prodrug constructs, e.g., comb-like or star-like, have been tested for the efficacious treatments of tumours [3]. The improved anticancer efficacy of mAb-containing polymer conjugates was observed *in vivo* in mice [15,16]. The proper selection of antibody can further enhance its biological effect and reduce drug cytotoxicity.

Recently, we described a new method for the preparation of highly effective star-shaped mAb-targeted polymer conjugates [17,18]. Semitelechelic HPMA copolymer molecules were linked to a central mAb via one-point attachments, forming a star-like structure with a relatively narrow molecular weight distribution. The synthesis was based on reducing the disulfide bridges of the mAb with dithiothreitol (DTT) under mild conditions followed by the one-point conjugation of semitelechelic polymers. This synthetic route preserves the binding ability of the targeting antibody to its specific cell receptors even after the conjugation with the polymer precursor.

One of the mAb candidates for targeted polymer prodrug therapy is the FDA-approved therapeutic monoclonal antibody rituximab (RTX), the first monoclonal antibody approved for clinical use in anti-cancer therapy. This antibody can be used in various diseases to reduce B-cell levels; however, RTX has primarily been used to treat non-Hodgkin’s B-cell lymphomas (B-NHL) [19,20]. RTX recognizes CD20 transmembrane proteins present on mature B-cells. After RTX binds to CD20 markers on the B-cell surface, multiple mechanisms are induced, leading to cell death [21]. The crosslinking of bound RTX induces the direct apoptosis of B-cells [22]. B-cells are also killed by natural killers (NK) through antibody-dependent cellular cytotoxicity (ADCC) or complement-dependent cytotoxicity (CDC), which is induced by the presence of the Fc part of RTX after binding [23].

In the present study, we describe and compare the synthesis and the properties of two different star-shaped polymer conjugates based on the HPMA copolymer: a dendrimer-derived HMW polymer conjugate for passive targeting and a star-shaped RTX-targeted conjugate for active targeting. Doxorubicin was bound to the polymer precursors through pH-sensitive, hydrolytically unstable hydrazone bonds tailored made for controlled drug release. Both polymer conjugates were evaluated for physicochemical and biological properties (*i.e.*, molecular weight; DOX *in vitro* release rate; *in vitro* cytotoxicity; mAb binding efficacy to CD20-positive and negative cells; and *in vivo* therapeutic efficacy). The pro-apoptotic and CD20-specific mAb RTX was selected as targeting moiety to evaluate potential use of RTX-targeted prodrug in the treatment of primary cell-based murine xenograft models of human diffuse large B-cell lymphomas, *i.e.*, possible synergistic outcome of pro-apoptotic effect and increased cytotoxicity of targeted polymer prodrug.

## 2. Results and Discussion

Recently, we described a new method for the synthesis of star-like polymer conjugates, based on dendrimer-containing polymer precursors [12] or on Ab-containing conjugates, using a mild reduction of cysteine units with DTT [18]. The DTT-based method overcomes the typical branching of reactions of a multivalent polymer with multivalent Abs used in earlier syntheses of Ab-targeted conjugates. Furthermore, this method does not significantly affect or restrict the Ab binding site as encountered in previously described mAb-targeted polymer-drug conjugates [24]. Moreover, the DTT-based method enables the consecutive reaction of Ab with the MI groups of semitelechelic HPMA-based polymers. Such polymer conjugates form a star structure with an Ab centre. In the present paper, we describe the synthesis and comparison of physico-chemical and biological properties of two different star-like polymer conjugates. The polymer conjugates are compared in terms of their synthesis, targeting ability, *in vitro* cytotoxicity, binding efficiency and *in vivo* anticancer efficacy. RTX was selected as the therapeutic candidate due to its pro-apoptotic properties and high specificity for the CD20 antigen. In addition, we investigated whether the RTX-targeted polymer-drug systems can combine both, pro-apoptotic signalling and increase of cytotoxicity by targeting the polymer-conjugate system to the vicinity of B-cell lymphomas, thus enhance the anti-lymphoma outcome. Serum immunoglobulin, flebogama, was used as a non-specific control antibody.

### 2.1. Synthesis of Semitelechelic Copolymers and Star Copolymers

**Figure 1:**
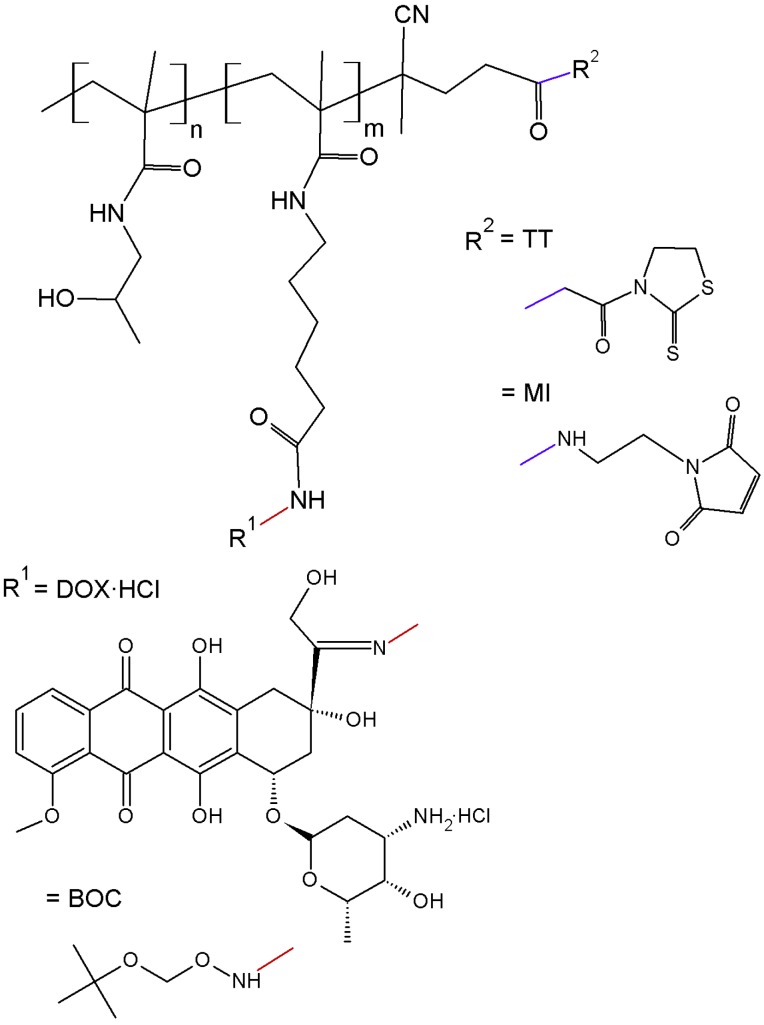
Scheme of semitelechelic polymer precursors used for star-shaped conjugates synthesis: polymer precursor 1 (TT and BOC), and polymer precursor 3 (MI and DOX).

The polymer precursor, semitelechelic copolymer **1** (Table 1, Figure 1), was prepared by the radical copolymerisation of HPMA with Ma-ah-NHNH-BOC and initiated with bifunctional initiator ABIK-TT. The copolymerisation with the initiator led to semitelechelic copolymers with chain-terminating reactive thiazolidine-2-thione (TT) groups with an end-chain functionality slightly greater than unity. The TT end-groups were easily converted to MI reactive groups suitable for selective reaction with thiol groups by aminolysis with *N*-(2-aminoethyl)maleimide. The transformation of the TT (polymer **1**) to MI end-groups (polymer **2**, Table 1) did not significantly alter the molecular weight and polydispersity of the polymer precursors, but the end-chain functionality slightly decreased closer to unity. The MI end-group was used for the one-point attachment of a semitelechelic copolymer to form a star-like structure. The molecular weight and polydispersity of polymer precursor **3** (Table 1), which was obtained by the reaction of DOX·HCl and polymer precursor **2**, slightly increased likely due to the side reaction of the MI end-groups with hydrazide groups.

**Figure 2:**
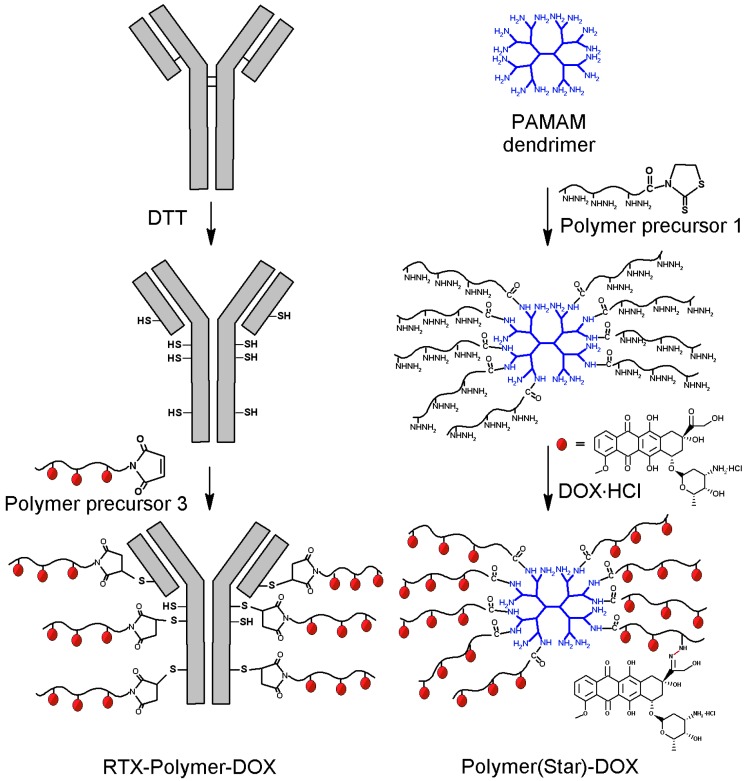
Schematic description of synthesis of star-like polymer-DOX conjugates: **left**—preparation of RTX-targeted polymer conjugate RTX-Pol-DOX; **right**—polymer conjugate Pol(Star)-DOX prepared by grafting PAMAM dendrimer.

The star dendrimer-based polymer precursor **5** was prepared by grafting via aminolytic reaction the semitelechelic polymer **1** onto the 2nd generation PAMAM dendrimer containing 16 amino groups (Figure 2). The product of the grafting reaction was a HMW polymer precursor **5** with a star structure (Table 1). This precursor is usable as a drug carrier for prolonged blood circulation and passive targeting to solid tumours. DOX was attached via hydrazone bond in methanol under acidic conditions to the linear polymer precursor **4** or the star-like polymer precursor **5** [6]. The attachment of DOX had no significant influence on the molecular weight or polydispersity of the polymer conjugates **Pol-DOX** and **Pol(Star)-DOX** (Table 2).

**Table 1:** Characteristics of polymer precursors.

Polymer Precursor	*M*_w_	*M*_w_/*M*_n_	Hydrazide Content (mol %)	End-Groups	*M*_n,T_^b^	*M*_n_/*M*_n,T_^c^	DOX (wt %)
**1**	25,000	1.51	-	TT	12,100	1.36	-
**2**	29,000	1.52	4.4	MI	18,500	1.03	-
**3**	44,900	2.26	-	MI	-	-	8.9
**4**	24,800	1.76	5.2	-	-	-	-
**5** ^a^	180,000	1.92	4.8	-	-	-	-

^a^ star-like dendrimed-based polymer precursor. ^b^ is number-average molecular weight calculated from the TT or MI group content. ^c^ is functionality of a polymer.

**Table 2:** Characteristics of polymer-drug and polymer-Ab conjugates.

Polymer Conjugate	*M*_w_	*M*_w_/*M*_n_	DOX (wt %)	Ab (wt %)
**Pol-DOX**	27,000	1.80	8.7	-
**Pol(Star)-DOX**	185,000	2.10	9.8	-
**RTX-Pol-DOX**	468,100	1.49	4.68	47.0 ^a^
**FLG-Pol-DOX**	427,900	1.83	4.40	47.5 ^b^
**RTX-Pol I**	281,700	1.33	-	46.6 ^a^
**RTX-Pol II**	299,600	1.26	-	56.8 ^a^
**RTX-Pol III**	328,600	1.32	-	64.5 ^a^

^a^ Conjugate containing mAb RTX; ^b^ Conjugate containing human polyclonal FLG.

### 2.2. Synthesis of RTX-Polymer Conjugates

For the preparation of the star Ab-based polymer conjugates, we used a synthetic strategy based on the mild reduction of the cysteine residues in the Ab with DTT (Figure 2). Generally, each modification of the Ab could lead to a partial loss of binding activity of the antibody to its antigen due to steric hindrance or to direct Ab modification within the binding site. We circumvented this issue by introducing thiol groups into the Ab by reducing with DTT the disulfide bonds located outside the antigen binding sites [25].

The reduction of inter-chain disulfides in the anti-CD20 Ab structure led to the formation of thiol groups; no significant changes in molecular weight, hydrodynamic radius or binding affinity to antigen were observed. On average, 9–10 thiol groups were introduced into one Ab, *i.e.*, an average of five reduced disulfide groups in each Ab, RTX or FLG. The reaction of the Ab-containing thiol groups with polymer precursors **2** and **3** containing end-chain MI groups enabled the attachment of 5–10 polymer chains to a single Ab molecule. To increase the cytotoxic efficacy of the proposed targeted polymer prodrug, *i.e.*, to deliver higher amounts of the cytotoxic drug per targeted polymer, we maximised the number of polymer chains attached to the mAb. However, the number of attached polymer chains is limited by the steric hindrance of the bulky polymers. Two different polymer precursors (polymers **2** and **3**) containing end-chain MI groups were attached to the SH groups of the modified Abs; drug-free polymer conjugates served as controls. A GPC analysis showed a narrow molecular weight distribution, indicating the formation of star-shaped structures without branching side reactions and a small content of unbound polymer in the crude product (less than 15 wt %). All the polymer-drug conjugates were purified from free DOX by gel permeation chromatography. Because of the solubility reason, RTX-containing polymer prodrugs were purified using PBS buffer as eluent and thus the RTX-containing prodrugs contained higher amount of free DOX (around 0.4%–0.7% of DOX) in comparison to the polymer conjugates **Pol-DOX** and **Pol(Star)-DOX** (around 0.1% of DOX), which were purified in organic solvent enabling more effective purification.

Both star-like polymer systems showed increased molecular weights, which promotes the EPR effect for the passive accumulation of nanomedicines within solid tumours. The molecular weight of the **RTX-Pol-DOX** conjugate was significantly higher than the drug-free **RTX-Pol** **I**-**III** conjugates due to the significantly higher molecular weight of the polymer precursor used in the preparation of DOX-containing conjugates. The molecular weights of the RTX-based star conjugates were approximately two times higher than those based on dendrimers. Similar observation was found for hydrodynamic radius of both star polymer conjugates. Hydrodynamic radius observed for **RTX-Pol-DOX** (24.3 nm) was significantly higher than that for **Pol(Star)-DOX** (13.2 nm). This enabled the prolonged circulation of the star-like polymer conjugates relative to lower molecular weight linear polymers.

### 2.3. Release of DOX from the Conjugates

**Figure 3:**
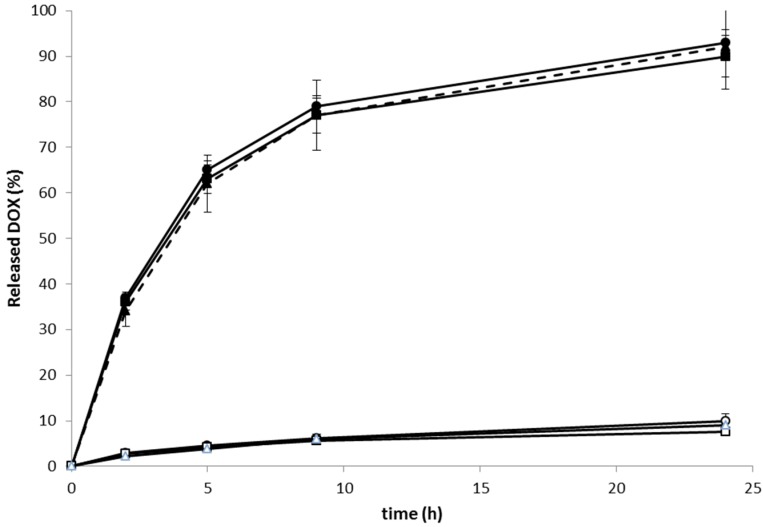
Release of DOX from linear, star and RTX containing polymer-DOX conjugates incubated in phosphate buffers at 37 °C: (● ──) **Pol-DOX**, pH 5; (▲ - - - ) **Pol(Star)-DOX**, pH 5; (■──) **RTX-Pol-DOX**, pH 5; (○──) **Pol-DOX**, pH 7.4; (∆──) **Pol(Star)-DOX**, pH 7.4; (□──) **RTX-Pol-DOX**, pH 7.4; *n* = 3.

The *in vitro* DOX release experiments decidedly showed that the linear, star and RTX containing conjugates with DOX attached via pH-labile hydrazone bonds (**Pol-DOX**, **Pol(Star)-DOX**, and **RTX-Pol-DOX**) were stable in buffer solutions mimicking physiological blood conditions (pH 7.4, 37 °C) (Figure 3), with up to 9% of DOX released within 24 h incubation.

On the contrary, the very fast release of approximately 90% of loaded DOX, was observed within 24 h of conjugate incubation in pH 5 buffers at 37 °C. The rate of drug release was almost independent of the structure of the polymer conjugate. The structure of polymer conjugate (e.g., linear, star or mAb-containing) or the type of used antibody (e.g., RTX or FLG) did not change the rate of release. The conjugation of a semitelechelic polymer-DOX conjugate to mAb did not change the drug release rate. Moreover, the results indicate that the Ab conjugates complied with the prerequisite criteria for an efficient anticancer prodrug, *i.e.*, stability in circulation and pH-controlled release of active drug after entering tumour cells/tissues.

### 2.4. In Vitro Cytotoxicity

The cytotoxicities of free DOX·HCl, **Pol-DOX**, **Pol(Star)-DOX**, **RTX-Pol-DOX** and **FLG-Pol-DOX** were determined in four B-cell lymphoma cell lines (UPF4D CD20^−^, UPF1H, Jeko-1 and SU-DHL-5) with different sensitivities to RTX and expression levels of CD20 (see Table 3). The inhibition of the proliferative capacity of the tested conjugates is expressed as IC_50_, *i.e.*, the concentration of doxorubicin required for 50% inhibition of cancer cell proliferation, as determined by the Alamar Blue viability reagent. The data presented in Table 3 show the different sensitivities of the tested cell lines to the original drug. The SU-DHL-5 cells were up to one order of magnitude more sensitive to DOX than the other three cell lines.

The results confirmed the earlier observed phenomenon of a decrease of one or two orders of magnitude in drug activity *in vitro* after binding to the polymer carrier. Using the Alamar Blue-based technique we have not observed significant changes in cytotoxicity for free RTX, FLG or **RTX-Pol I** (data not shown) in comparison with untreated cells in used concentration range in this study. We found no significant differences in the cytotoxic activities of linear and HMW star polymer conjugates (compare **Pol-DOX** and **Pol(Star)-DOX** in Table 3). The cytotoxic activities of both polymers are likely correlated with the rate of release of DOX from the polymer carriers; the molecular weights and structures of the carriers did not influence the cytotoxicity *in vitro*. Indeed, higher anti-tumour activity *in vivo* is assumed for the HMW star polymer **RTX-Pol-DOX**. No significant effect of CD20-specific targeting was observed in any of the tested cell lines. Both antibody-containing **RTX-Pol-DOX** and **FLG-Pol-DOX** conjugates had similar IC_50_ values for all tested cell lines, independent of the expression of CD20 or sensitivity to RTX. We recently described similar findings for other CD20 mAbs [18]. The use of CD20 mAbs for targeting clearly enhanced the normally low cytotoxic activity of conjugates containing DOX bound through enzymatically-degradable spacers with slow drug release profiles. However, no positive effects were observed for the highly active conjugates where DOX was bound through pH-labile hydrazone bonds and rate of the drug release was several times higher than in the case of enzymatically degradable spacer. The effect of fast release of drug in conjugates with hydrazone-bound DOX, drug is released even in extracellular space, was in an excess of effect of CD20-specific targeting.

**Table 3:** Cytostatic activity ^a^ of polymer conjugates.

Conjugate	UPF1H (RTX s.;CD20^+^) ^b^	UPF4D (RTX ns.; CD20^−^) ^b^	Jeko-1 (RTX ns.; CD20^+^) ^b^	SU-DHL-5 (RTX s.; CD20^+^) ^b^
**DOX·HCl**	0.020 ± 0.006	0.016 ± 0.004	0.090 ± 0.027	0.004 ± 0.002
**Pol-DOX**	1.001 ± 0.552	1.041 ± 0.576	1.105 ± 0.490	0.094 ± 0.081
**Pol(Star)-DOX**	0.724 ± 0.710	1.073 ± 0.745	1.176 ± 0.733	0.046 ± 0.019
**RTX-Pol-DOX**	0.440 ± 0.075	0.336 ± 0.046	0.285 ± 0.065	0.040 ± 0.014
**FLG-Pol-DOX**	0.446 ± 0.150	0.279 ± 0.083	0.361 ± 0.047	0.047 ± 0.018

^a^ IC_50_ in μg/mL DOX (Alamar Blue); ^b^ Sensitivity to RTX: s. is sensitive, ns. is nonsensitive; CD20^+^ or CD20^−^ means expression of CD20.

The slightly higher cytotoxicities of the Ab-containing conjugates **RTX-Pol-DOX** and **FLG-Pol-DOX** relative to **Pol-DOX** and **Pol(Star)-DOX** could be ascribed to two independent mechanisms. First, the Ab-containing polymer conjugates contain higher amounts of free DOX as discussed above, likely due to the different syntheses and purification for the Ab-containing and the non-containing polymer conjugates. Second, the direct effect of the Ab in polymer conjugates may play a role in increasing the cytotoxicity.

### 2.5. In Vitro Study of RTX Binding Efficacy to CD20 Antigen

**Figure 4:**
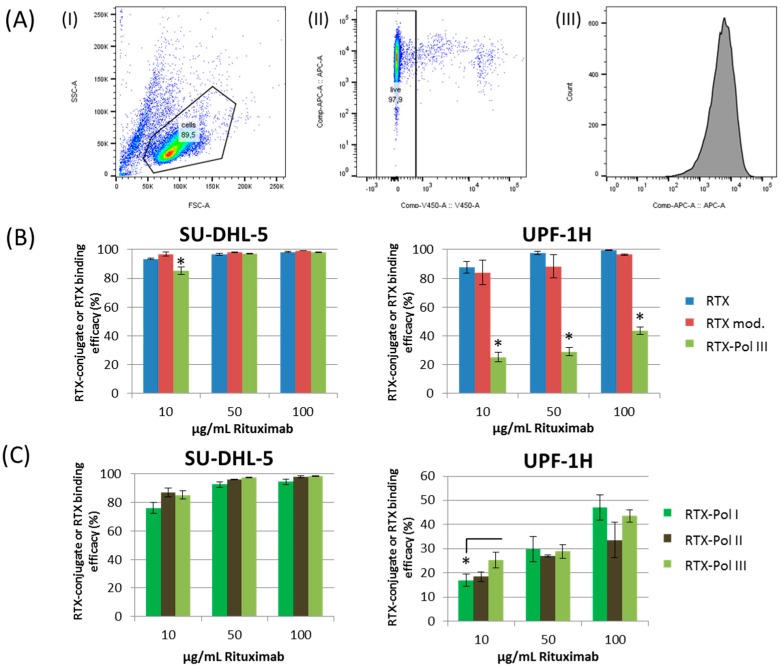
RTX and RTX-targeted conjugate **RTX-Pol I**-**III** binding activity to SU-DHL-5 and UPF1H cell lines measured by flow cytometry using nonspecific binding assay. (**A**) Gating strategy used for analysis: (I) cell population, (II) live cells, (III) histogram with APC-fluorescence; (**B**) binding efficacy of RTX, RTX mod. (RTX reduced by DTT) and **RTX-Pol III**; (**C**) binding efficacy of three different RTX-polymer conjugates: **RTX-Pol I** (46.6% RTX), **RTX-Pol II** (56.8% RTX) and **RTX-Pol III** (64.5% RTX). Significant differences *p* < 0.05 are labelled with asterisks.

The binding activities of RTX-containing (**RTX-Pol I**-**III**) conjugates, RTX and DTT-modified RTX (RTX mod.) on the CD20 receptor on the selected cell lines were compared by competitive binding assay. Cells were incubated with three concentrations (10, 50 and 100 µg RTX/mL) of RTX, RTX mod., **RTX-Pol I**-**III** and polymer precursor **4**. Figure 4a shows the gating strategy used for the evaluation of the data. The SU-DHL-5 cell line showed similar binding efficacies for all tested conjugates, **RTX-Pol I**-**III** and RTX (see Figure 4b,c), excluding a small decrease in the smallest RTX concentration for all tested RTX-polymer conjugates. Comparable results were also observed for the Jeko-1 cell line (data not shown). The RTX and DTT-modified RTX did not show significant differences in binding efficacy to surface CD20 receptors for the SU-DHL-5 and UPF1H cell lines (Figure 4b). A significantly decreased binding efficacy of **RTX-Pol III** in the UPF1H cell line was observed, ranging from 30% to 40% of the original activity of RTX for the tested concentrations. The decreased binding efficacy of **RTX-Pol III** to the UPF1H cell line was likely attributed to the cell line origin. The UPF1H cell line was established from the patient after RTX therapy, which can cause changes in expression of the epitopes for the CD20 receptor and, moreover, result in the modification of the epitopes for the recognition of Ab. This modification is significant due to the increased steric hindrance on the binding efficacy of bulk polymer-RTX conjugates.

We primarily observed negligible differences in the binding efficacy between the conjugates with different amounts of RTX. However, only in the lowest tested concentrations, a slightly lower binding efficacy for **RTX-Pol I** with lower RTX content was observed (Figure 4c). No differences in the binding efficacy between conjugates containing DOX (**RTX-Pol-DOX**) or non-containing DOX (**RTX-Pol I**) were observed, indicating that the presence of the drug did not influence the binding efficacy to the antigen (data not shown). No binding activity was detected for the UPF4D CD20^−^ negative control cell line for all tested samples and RTX (data not shown). Overall, we have shown that the modification of RTX with semitelechelic hydrophilic copolymers slightly decreased the binding activity to the CD20 receptor. The decrease in binding activity was strongly dependent on the cell line origin being highly pronounced for UPF1H cell line.

### 2.6. Induction of Apoptosis

**Figure 5:**
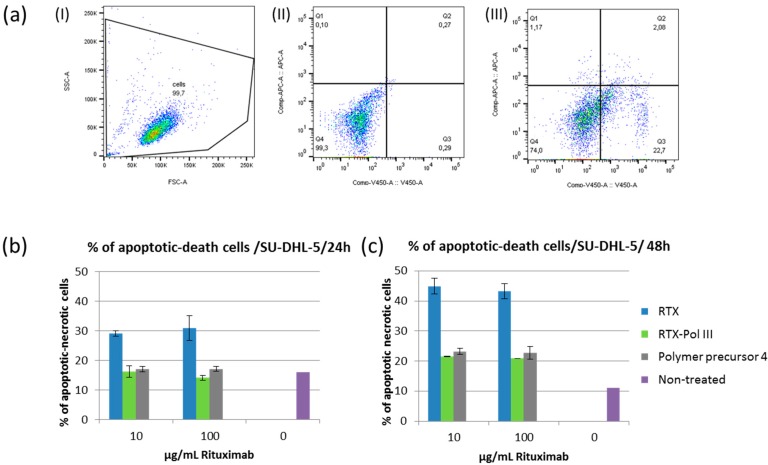
Percentage of apoptotic or dead cells after incubation with RTX, **RTX-Pol III** or polymer precursor **4**; (**a**) gating strategy used for analysis: (I) cell population, (II) non-treated cells, (III) RTX-treated cells (quadrants were based on the intensity of annexin-APC and Sytox Blue-V450); (**b**) cell death changes after 24 h of incubation; (**c**) cell death changes after 48 h of incubation.

We evaluated the cytotoxic effects of RTX and RTX-polymer conjugates *in vitro* using different B-NHL cell lines. We incubated RTX-sensitive (SU-DHL-5) and RTX-non-sensitive (Jeko-1) cell lines for 24 h and 48 h with RTX, **RTX-Pol III** or polymer precursor **4** at 10 or 100 µg/mL RTX final concentration or appropriate amounts of polymer. Pro-apoptotic changes and dead cells were analysed using Annexin-V and Sytox Blue staining, respectively. The treatments with RTX and **RTX-Pol III** did not cause any apoptotic changes for Jeko-1 cells, which are not sensitive to RTX (data not shown). Figure 5b,c show cell deaths of 30%–35% and 45% of cells in the samples treated with RTX for 24 h and 48 h, respectively. Incubation with **RTX-Pol III** and polymer precursor after 48 h caused significantly higher cell death (20%–25%) when compared with controls (approximately 10% cell death) but significantly lower cell death than that of RTX. The effect was similar for polymer conjugates with or without RTX. These results showed the small effects of the polymer carrier after 48 h incubation with the cells, which were negligible when compared with the effects of RTX. The results show that the RTX-polymer conjugates did not induce cell death changes as free RTX.

### 2.7. In Vivo Experimental Therapy

**Figure 6:**
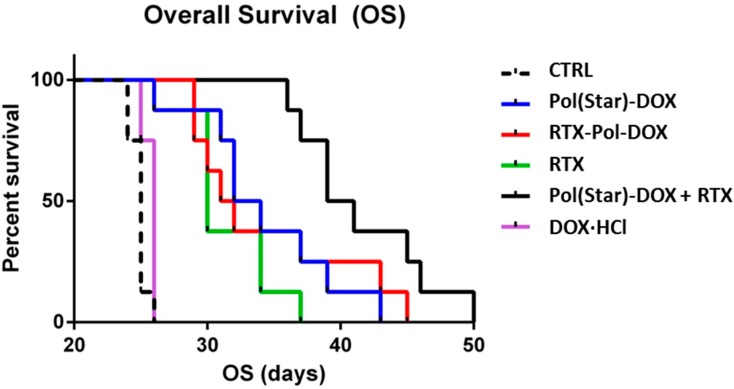
Comparison of anti-tumour activities of conjugate **Pol(Star)-DOX**, **RTX-Pol-DOX**, RTX, DOX·HCl and combination of RTX and **Pol(Star)-DOX** in human DLBCL lymphoma model (CTRL, non-treated). Overall survival was monitored.

The median overall survivals (OS) of the treatment cohorts were 25, 30, 33, 31.5, 40 and 25 days for the untreated animals (CTRL), RTX-treated mice, **Pol(Star)-DOX**-treated mice, **RTX-Pol-DOX**-treated mice, **Pol(Star)-DOX** + RTX-treated mice and doxorubicin-treated mice, respectively (Figure 6). Doxorubicin administered at the maximum tolerated dose (1.25 mg/kg, corresponding to 25 mcg/dose) did not prolong the survival rate when compared with untreated animals. Single-agent RTX significantly prolonged the survival rate of the treated mice (*p* = 0.003). Interestingly, there was no significant difference in the OS between the **Pol(Star)-DOX**-treated mice and the **RTX-Pol-DOX**-treated mice (*p* = 0.713). In contrast, there was a strong trend towards an improved OS in the cohort treated with the combination of **Pol(Star)-DOX** and rituximab when compared with the **RTX-Pol-DOX** conjugate (*p* = 0.0513). The combination of the star polymer **Pol(Star)-DOX** and rituximab led to significantly improved survival when compared with mice treated with single-agent **Pol(Star)-DOX** (*p* = 0.017). This appeared to be the most effective anti-lymphoma treatment strategy, at least for the KTC primary cell-based model of treatment-refractory (CD20^+^) DLBCL.

Based on these data, we conclude that targeting of polymer-bound anthracyclines with CD20 monoclonal antibody RTX does not prolong overall survival when compared with **Pol(Star)-DOX**. However, the synergistic effects of a combination of star **Pol(Star)-DOX** and RTX was profound and led to the optimal therapy outcome. It seems that in the star polymer and RTX combination therapy, both components maintain their original activity (*i.e*., the pro-apoptotic effects of RTX and cytotoxic activities of the star polymer conjugate), which leads to an enhanced therapy effect. Unfortunately, the modification of RTX with polymer chains resulted in the decreased pro-apoptotic activity of RTX, as discussed above, and likely also led to the simultaneous decrease of released drug because of the non-internalising mechanism for RTX. The results of the flow cytometry analyses further indicate the potential loss of the immunological properties of the polymer-bound RTX (*i.e.*, a decreased capacity to bind CD20). The results suggest that the anti-CD20 antibody rituximab does not represent an ideal mAb for targeting polymer prodrugs, especially in such patients who relapse after the failure of rituximab-based front-line therapies. The use of other mAbs, the binding of which would ideally result in the internalisation of the mAb-antigen complexes, may be alternatives to RTX for more effective polymer-prodrug targeting strategies.

## 3. Experimental Section

1-Aminopropan-2-ol, methacryloyl chloride, 2,2′-azobis(isobutyronitrile) (AIBN), 3,3'-[azobis(4-cyano-4-methyl-1-oxobutan-4,1-diyl)]bis(thiazolidine-2-thione) (ABIK-TT), 6-aminohexanoic acid (AH), dimethylformamide (DMF), phthalaldehyde (OPA), *N*-ethyldiisopropylamine, *N*-(2-aminoethyl)maleimide trifluoroacetate, 5,5′-disulfanylbis(2-nitrobenzoic acid), cysteine, dithiothreitol (DTT), dimethyl sulfoxide (DMSO), tert-butyl carbazate, trifluoroacetic acid (TFA) and doxorubicin hydrochloride (DOX·HCl) were purchased from Sigma-Aldirch (Prague, Czech Republic). 2,4,6-Trinitrobenzene-1-sulfonic acid (TNBSA) was purchased from Serva (Heidelberg, Germany). *N*-(*tert*-butoxycarbonylaminopropyl)-methacrylamide was purchased from Polysciences (Niles, IL, USA). Serum immunoglobulin, monoclonal antibody rituximab (MabThera^®^, Roche, Basel, Switzerland) and polyclonal antibody flebogamma (FLG) (Flebogamma^®^, Grifols, Barcelona, Spain) were purified from excipients (e.g., glukose, NaCl, glycin) before conjugation by filtration using a Amicon^®^ Ultra centrifugal filter (Millipore, Carrigtwohill, Ireland) units with cut-off 30,000 Da and distilled water as solvent.

### 3.1. Synthesis of Monomers

*N*-(2-Hydroxypropyl)methacrylamide (HPMA) was synthesized as described in [5]. M.P. 69–70 °C; elemental analysis: calc. C 58.72%, H 9.15%, N 9.78%; found C 58.98%, H 9.18%, N 9.82%. *N*-(*tert*-butoxycarbonyl)-*N'*-(6-methacrylamidohexanoyl)hydrazine (Ma-ah-NHNH-BOC) was prepared in two-step synthesis as described in [5]. M.P. 110–114 °C, elemental analysis: calc. C 57.70%, H 8.33%, N 13.46%; found C 57.96%, H 8.64%, N 13.25%. Purity of all monomers was examined by HPLC (Shimadzu 10VP, Kyoto, Japan) using a C_18_ Chromolith Performance RP-18e reverse-phase column (4.6 × 100 mm) with diode array detection, eluent water-acetonitrile with gradient 5–95 vol % acetonitrile, 0.1% TFA, flow-rate 1 mL/min.

### 3.2. Synthesis of Polymer Precursors and Polymer-Drug Conjugates

Semitelechelic polymer precursor (polymer precursor **1**, Table 1) containing BOC-protected hydrazide groups was prepared by free radical copolymerization as previously described [17]. The polymerization was carried out at 60 °C for 6 h. The polymer was isolated by precipitation into an acetone/diethyl ether mixture (1:1), purified by precipitation in methanol, filtered and dried in vacuum. The TT group content was determined spectrophotometrically on a Helios α (Thermo Fisher Scientific, Waltham, MA, USA) spectrophotometer (ε_305_ = 10700 L·mol^−1^·cm^−1^ in methanol [26]).

The end-chain reactive maleimide (MI) group was introduced into polymer precursor **1** by the aminolytic reaction of *N*-(2-aminoethyl)maleimide with the TT group. Briefly, *N*-(2-aminoethyl)maleimide trifluoroacetate (45 mg, 0.177 mM) was dissolved in DMF (1.5 mL); *N*-ethyldiisopropylamine (58 µL) was added under stirring. After 2 h, the reaction mixture was diluted to 9 mL with methanol, and low-molecular-weight impurities were removed by gel filtration using a Sephadex LH-20 column with methanol elution. The polymer fractions were collected, the purified polymer precursor was isolated by precipitation into ethyl acetate, and the BOC-protecting groups were removed with concentrated TFA to yield a semitelechelic polymer precursor **2** (Table 1).

Copolymer of HPMA with Ma-ah-NHNH_2_ (polymer **4**, Table 1) was prepared by a previously described reaction [27]. The star polymer precursor **5** was prepared by grafting the reactive semitelechelic HPMA copolymer precursor **1** onto the 2nd generation (G2) PAMAM dendrimer containing 16 terminal reactive amino groups and a diaminobutane core. Briefly, polymer precursor **1** (354 mg; 0.03 mM TT groups) was dissolved in methanol (9 mL) and added into a stirring solution of PAMAM dendrimer (9 mg) in methanol (3.1 mL). After 2 h, the reaction was terminated by adding 1-aminopropan-2-ol (5 μL). Low-molecular-weight (LMW) impurities were removed by gel filtration (Sephadex LH-20 in methanol solvent). The polymer-modified dendrimer was isolated by precipitation in ethyl acetate. The free hydrazide groups required for DOX attachment were obtained in polymer precursor **5** by removing the protective BOC groups from the hydrazides with concentrated TFA.

Semitelechelic polymer precursor **3** and the polymer conjugates **Pol-DOX** and **Pol(Star)-DOX** (Table 2) were similarly prepared by reacting the polymer precursors **2**, **4** or **5** containing hydrazide groups with DOX·HCl in methanol and acetic acid as previously described [28]. 

### 3.3. Synthesis of Antibody-Polymer-DOX Conjugates

MAb-containing polymer conjugates, **RTX-Pol-DOX**, **FLG-Pol-DOX**, and **RTX-Pol I**-**III** (Table 2), were prepared from polymer precursor **2** or DOX-containing semitelechelic polymer precursor **3** by conjugation with monoclonal (RTX) or polyclonal (FLG) antibody reduced by DTT as previously described [18].

Briefly polymer precursor **3** (290 mg) and reduced RTX or FLG (220 mg) were dissolved to a final volume of 22.4 mL in phosphate buffer (pH 7.2, 0.1 M NaCl, 1 mM EDTA, bubbled with argon). Semitelechelic polymers with MI end groups reacted with the SH group in the mAbs to form covalent thioester bonds. The polymer antibody conjugate were desalted by chromatography on a G-25 column and lyophilized.

### 3.4. Purification and Characterization of Conjugates

All polymer mAb conjugates were characterized and tested for free polymer, drug or mAb contents using a HPLC equipped with UV (Shimadzu, Kyoto, Japan), refractive index (Optilab^®^-rEX, Wyatt Technology Corp., Santa Barbara, CA, USA) and multi-angle light scattering (DAWN EOS detector, Wyatt Technology Co., Santa Barbara, CA, USA) using 0.3 M acetate buffer (pH 6.5) and a Superose™ 6 column. The mAb content in the conjugates was estimated by amino acid analysis (precolumn OPA derivatisation, Shimadzu, Kyoto, Japan). The DOX content was estimated by UV spectrophotometry [29]. The hydrazide groups content was determined by a modified TNBSA assay as previously described [6]. The MI group content in the polymer precursors was determined by a modified Ellman’s assay as the difference between cysteine concentrations before and after reaction with the MI groups of the polymer [15]. The amount of introduced thiol groups was determined by reaction with Ellman’s reagent [30]. The dynamic (DLS) light scattering of aqueous conjugates solutions was measured at the scattering angle 173 ° on a Zetasizer Nano ZS, Model ZEN3600 (Malvern, Worcestershire, UK). The hydrodynamic radius (Rh) was determined by the DTS (Nano) program (Malvern, Worcestershire, UK, 7.10).

### 3.5. In Vitro Release of Doxorubicin from Polymer Drug Conjugates

The stability of the hydrazone bonds and the release of DOX from polymer drug conjugates (polymer concentration equivalent to 0.5 mM DOX) were investigated in phosphate buffered saline at pH 5.0 or 7.4 (0.1 M phosphate buffer with 0.05 M NaCl) at 37 °C. Released DOX were extracted into an organic solvent and analysed by HPLC as previously described [7]. All drug-release data are expressed as the amount of soluble drug relative to the total drug content in the conjugates. All experiments were carried out in triplicate. 

### 3.6. Cell Lines

UPF4D and UPF1H cell lines (both CD20 positive) were derived by Dr Klener’s group at the Institute of Pathological Physiology of the Charles University in Prague from patients with treatment-refractory diffuse large B-cell lymphoma (DLBCL) and mantle cell lymphoma (MCL), respectively. In both cases, the cell lines were established after rituximab-based treatments. UPF4D CD20^−^ cells were obtained by a long-term culture (3 months) of the original UPF4D cell line. The reason for the spontaneous loss of CD20 expression remained elusive. The sequencing of IGHV confirmed the clonal identity of the UPF4D and UPF1H cell lines with the primary DLBCL and MCL cells, respectively (data not shown). SU-DHL-5 and Jeko-1 cell lines were obtained from the DSMZ (German Collection of Microorganisms and Cell Cultures, Braunschweig, Germany). The cells were cultivated in RPMI-1640 medium (Thermo Scientific, Prague, Czech Republic) supplemented with heat-inactivated 10% FBS for SU-DHL-5 and Jeko-1 cells or 15% FBS for UPF1H and UPF4D cells and penicillin (100 U/mL) and streptomycin (100 µg/mL).

### 3.7. Primary DLBCL-Based Murine Xenograft Model Establishment

Primary DLBCL cells were obtained from the cervical lymph nodes of a patient with treatment-refractory diffuse-large B-cell lymphoma. The sample was obtained after informed consent according to the Declaration of Helsinki. The lymph node sample was filtered through a 45 µm nylon mesh. The extracted primary cells were subcutaneously injected into the right flank of NSG mice (*n* = 6). After the mice developed s.c. tumours, the animals were euthanised, the tumours excised, passed through a 45 µm nylon mesh and frozen in aliquots (designated as KTC). The cells were subject to multi-colour FISH analysis and flow cytometry analysis (for 60 CD antigens). Both methods confirmed the identity of the primary DLBCL cells isolated from the lymph node. It must be emphasized that KTC cells do not grow *in vitro* and can only be propagated by serial transplantations from donor to recipient NSG mice. For the purpose of this study, one aliquot of KTC cells was thawed and s.c. injected into three NSG mice. After the mice developed s.c. tumours (approximately 2 cm in the largest diameter), the mice were euthanised, and the cells (processed as described above) were injected into fifty-six female NSG mice (each animal received 10 × 10^6^ cells in 300 µL of PBS into the right flank). The day of injection was designated as D + 1.

### 3.8. In Vitro Cell Viability Assay

Ten thousand cells were seeded in 100 µL of media per well in 96-well flat-bottom plates (TPP, Sigma-Aldrich, Prague, Czech Republic) 24 h before adding the polymer precursors, polymer conjugates, antibodies or DOX·HCl. The range of concentrations used was 0.02–10 µg/mL DOX for conjugates and 0.015–5 µg/mL for DOX·HCl. The polymer precursors and antibodies were measured in concentration equivalents to that used for the polymer-DOX conjugates. The cells were cultivated for 72 h in 5% CO_2_ at 37 °C. Subsequently, 10 µL of AlamarBlue^®^ cell viability reagent (Thermo Scientific, Prague, Czech Republic) was added to each well, and the cultures were incubated for 4 h at 37 °C. In this assay, the active component of the AlamarBlue reagent resazurin is reduced to the highly fluorescent compound resorufin only in the viable cells. Fluorescence was measured by a Synergy Neo plate reader (Bio-Tek, Prague, Czech Republic) at 570 nm excitation and 600 nm emission. As a control, non-treated cells were used. All samples were measured in duplicate in three independent measurements. 

### 3.9. In Vitro Study of RTX Binding Efficacy to Its Antigen (CD20)

The binding activities of the RTX-containing (**RTX-Pol I**-**III**) conjugates in comparison with the RTX and DTT-modified RTX (RTX mod.) to cell surface epitopes of lymphoma cells (CD20) were evaluated by competitive binding assays. This is indirect methods based on the measurement of decreased fluorescence (by FACS) of anti-CD20-APC antibodies bound to the cell antigens with or without pretreatment with RTX or RTX-containing conjugates. 

Briefly, cells were washed with 0.5% BSA-PBS, and 2 × 10^5^ cells in 50 μL volumes were incubated in duplicate for 30–40 min with RTX and RTX-targeted polymer conjugates. The final concentrations of RTX were 10, 50 or 100 μg RTX/mL. The samples were then washed with 0.5% BSA-PBS, resuspended in 50 μL of 0.5% BSA-PBS and incubated with saturation concentrations (2.5 μL for SU-DHL-5, Jeko-1, and UPF4D and 5 μL for UPF1H) of competitive mouse anti-human CD20 monoclonal antibody conjugated with APC (anti-CD20-APC) (Exbio, Prague, Czech Republic) for 30–40 min. Afterwards, the cells were washed with 0.5% BSA-PBS and diluted in 0.5 mL 0.5% BSA-PBS with 1 μg/mL Sytox Blue reagent (Thermo Scientific, Prague, Czech Republic) for viability counts. Samples were analysed using FACS Verse (Becton Dickinson, Franklin Lakes, NJ, USA).

The binding efficacies of the RTX-targeted conjugates or RTX alone were calculated as the differences between the fluorescent intensities of the CD20-APC-marked original cells and the CD20-APC-marked cells after treatment with the RTX-targeted polymer conjugates or RTX alone. Significant differences were performed using GraphPad Prism (La Jolla, CA, USA, 5.5).

### 3.10. Determining Conjugate Ability to Induce Apoptotic Changes

The RTX-targeted conjugates were assessed for their ability to induce direct apoptotic changes. Samples were analysed by flow cytometry using Annexing V-APC and Sytox Blue labelling (Thermo Scientific, Prague, Czech Republic). SU-DHL-5 and Jeko-1 cells were seeded at 1.2 × 10^5^ cells/2 mL RPMI medium for 24 h in 12-well plates. The RTX-targeted conjugate, free RTX or polymer precursor at final concentrations of 10, 50 or 100 μg RTX/mL or the corresponding concentrations of polymer precursors were added. Cells were incubated for 24 h or 48 h. Cells were stained by Annexin-V-APC to evaluate apoptotic changes and Sytox Blue to determine cell viability. Samples were analysed using FACS Canto II (Becton Dickinson, Franklin Lakes, NJ, USA).

### 3.11. Immunodeficient Mice

NOD·Cg-Prkdc^scid^ Il2rg^tm1Wjl^/SzJ mice (referred to as NSG mice) were purchased from The Jackson Laboratory (Bar Harbor, ME, USA). All animals were housed and maintained in a pathogen-free environment in individually-ventilated cages and provided with sterilised food and water. The experimental design was approved by the institutional animal care and use committee.

### 3.12. Experimental Therapy

Mice (average weight 20 g) were stratified into seven treatment cohorts, each comprising eight animals as follows: untreated animals (CTRL), rituximab-treated animals (RTX), **Pol(Star)-DOX**-treated animals, **RTX-Pol-DOX**-treated animals, two cohorts with conventional doxorubicin-treated animals (DOX·HCl) and one combinatorial cohort (**Pol(Star)-DOX** + RTX). The doses of the polymer-bound doxorubicin agents **Pol(Star)-DOX** and **RTX-Pol-DOX** were calculated according to their respective contents of doxorubicin; the mice received 5 mg/kg/dose doxorubicin (approximately corresponding to a flank dose of 100 mcg of conventional doxorubicin). Conventional doxorubicin (obtained from TEVA) was given under two dosing schemes, 2.5 mg/kg/dose and 1.25 mg/kg/dose, corresponding to net doses of 50 and 25 mcg per dose. However, all mice treated with 2.5 mg/kg DOX·HCl died due to toxicity and were not included in the data analysis (Figure 6). Rituximab was given at a dose that corresponded to that obtained in **RTX-Pol-DOX**. Therapy was initiated at day 5 (D + 5). Mice received a total of 3 cycles of therapy in 7-day intervals (D + 5, D + 12, D + 19). All agents were administered intravenously (i.v.) with the exception of RTX, which was given s.c. (both in single-agent and combinatorial treatment cohorts). Untreated mice received PBS only. Animals were euthanised after they developed hind-leg paralysis or a general inability to thrive (slow movement, tremor, tachypnea, progressive wasting and other symptoms of advanced disease). Differences in survival rates between treatment groups were evaluated using Kaplan-Meier survival estimates with GraphPad Prism software. Statistical analyses were performed using GraphPad Prism.

## 4. Conclusions

We described the synthesis and characterisation of biological properties of non-targeted or anti-CD20 monoclonal antibody-targeted star-shaped HMW polymer-doxorubicin conjugates. Non-targeted polymer conjugates were prepared as long-circulating HMW polymer prodrugs with dendrimer core suitable for prolonged circulation in the body and possible passive accumulation in tumour masses via the EPR effect. We tested the possibility of active targeting by conjugation with the anti-CD20 monoclonal antibody rituximab. When compared with the original, unmodified monoclonal antibody, the modification of rituximab with semitelechelic hydrophilic copolymers lead to decreased immunologic and functional properties of the antibody, including the above-described slight decreased binding activity of rituximab towards the CD20 antigen or the inhibition of its pro-apoptotic activity. Thus, the potential synergistic effect of the pro-apoptotic effect of RTX and increased cytotoxicity of targeted polymer conjugate was not observed. *In vivo* experiments using primary cell-based murine xenograft models of human diffuse large B-cell lymphoma confirmed that the targeting of polymer-bound anthracyclines with RTX did not significantly prolong the overall survival when compared with the corresponding non-targeted star polymer. However, the combination of rituximab and non-targeted star-shaped polymer prodrug provided significantly prolonged overall survival and was the best therapy strategy. We conclude that the anti-CD20 antibody rituximab is not an ideal mAb for targeting polymer prodrugs especially in term of anti-lymphoma efficacy, but the combination therapy using separately the long-circulating polymer prodrug and rituximab could improve therapeutic results in treatment of highly aggressive lymphoma.
